# Application of Digital Health Interventions for Cognitive Management in Children With Cancer: Scoping Review

**DOI:** 10.2196/104881

**Published:** 2026-11-05

**Authors:** Tao Liu, Saad Saif, MaoTing Tang, Lin Mo

**Affiliations:** 1 Department of Outpatient, Children’s Hospital of Chongqing Medical University, National Clinical Research Center for Children and Adolescents' Health and Diseases, Ministry of Education Key Laboratory of Child Development and Disorders, International Science and Technology Cooperation Base of Child Development and Critical Disorders, Chongqing Key Laboratory of Child Neurodevelopment and Cognitive Disorders, No.136, Second Road, Yuzhong District, Chongqing, 404100, China, 86 13452889397; 2 School of Nursing, Chongqing Medical University, Chongqing, China

**Keywords:** pediatric cancer, cognitive late effects, cognitive management, digital health, scoping review

## Abstract

**Background:**

Advances in the diagnosis and treatment of childhood cancer have substantially improved survival, resulting in a growing population of survivors of childhood cancer who may experience long-term treatment-related sequelae. Cognitive late effects are an important long-term consequence of childhood cancer and can affect cognitive functioning, psychosocial well-being, and quality of life. Digital health interventions offer accessible and flexible approaches to managing cognitive difficulties among children with cancer. However, the existing evidence has not yet been comprehensively synthesized.

**Objective:**

This study aimed to map and synthesize the existing evidence on digital health interventions for cognitive management among children with cancer; characterize intervention modalities, delivery approaches, and functional components; and identify evidence gaps and priorities for future research.

**Methods:**

This scoping review was conducted in accordance with the Arksey and O’Malley framework and the PRISMA-ScR (Preferred Reporting Items for Systematic Reviews and Meta-Analyses Extension for Scoping Reviews) checklist. Six academic databases, including PubMed, Embase (Ovid), CINAHL, PsycINFO, Web of Science, and the Cochrane Library, were systematically searched for relevant studies published between January 2010 and August 2026. Eligibility criteria were developed using the population-concept-context framework. Two reviewers independently performed study screening and data extraction. The methodological quality of included studies was assessed using the Mixed Methods Appraisal Tool. Screening was performed independently at the title/abstract and full-text stages, with disagreements resolved through discussion and, when necessary, consultation with a third reviewer. Descriptive statistics and thematic content analysis were used to synthesize the findings.

**Results:**

A total of 5371 records were identified through the literature search. Following screening, 19 publications representing 14 independent study samples and involving 566 children with cancer were included. Four digital health modalities were identified: computer-based interventions, internet-based interventions, mobile apps, and neurofeedback training. Computer-based interventions were the predominant modality. Across these modalities, working memory and attention were the most frequently targeted cognitive domains, while feasibility, acceptability, and adherence were the most commonly assessed implementation-related outcomes. However, relatively few interventions incorporated coach support, telephone-based communication, or educational components, and none evaluated economic outcomes.

**Conclusions:**

Although digital health interventions show promise for supporting cognitive function in children with cancer, substantial gaps remain in the current evidence. This scoping review mapped the field according to intervention modalities, delivery approaches, and functional components, rather than focusing solely on the efficacy of individual technologies. Future research should prioritize the development of multidimensional, personalized, and functionally integrated digital health interventions that combine cognitive training with supportive care, education, monitoring, and communication. Multicenter randomized controlled trials should subsequently evaluate these interventions using clinically meaningful outcomes, with comprehensive assessments of safety, adherence, implementation, and health economic outcomes to establish their effectiveness, feasibility, and sustainability.

## Introduction

Childhood cancer remains a major global pediatric health challenge, with recent estimates indicating that approximately 377,000 children are newly diagnosed worldwide each year [1]. Advances in diagnostic approaches, risk-adapted multimodal therapy, and supportive care have substantially improved survival outcomes, particularly in high-income countries where 5-year survival rates now exceed 80% [2]. This progress has led to a rapidly expanding population of survivors of childhood cancer and has shifted clinical and research priorities from survival alone toward the identification, prevention, and management of long-term treatment-related sequelae [3]. Among these survivorship concerns, cognitive late effects are some of the most clinically significant and functionally disabling outcomes [4]. Evidence suggests that approximately 40% to 60% of survivors of childhood cancer experience cognitive late effects, with impairments most commonly observed in attention, executive function, processing speed, and other higher-order cognitive domains [5-7]. These difficulties may emerge during treatment, progress over time, or become more apparent as survivors encounter increasing academic and social demands. Persisting into adolescence and adulthood, neurocognitive impairments can adversely affect educational achievement, employment opportunities, psychological well-being, and social participation [8,9]. Accordingly, cognitive late effects represent not only a major survivorship concern for survivors of childhood cancer and their families but also an important public health issue with long-term implications for health care systems and society [10-12].

The management of cognitive late effects in survivors of childhood cancer remains a substantial challenge in clinical practice [13,14]. Cognitive management can be conceptualized as a comprehensive approach involving cognitive training, cognitive rehabilitation, cognitive monitoring, and supportive strategies aimed at improving or maintaining cognitive function [15,16]. Previous studies have shown that pharmacological treatments, physical exercise, and cognitive training may contribute to improvements in cognitive outcomes [17,18]. However, the effectiveness, accessibility, and adherence of these interventions are often constrained by medication-related adverse effects, developmental considerations, age-specific feasibility, and the need for sustained engagement. These challenges are further amplified in resource-limited settings and during intervals between routine clinical follow-up visits, highlighting the need for accessible, scalable, and continuous approaches to cognitive management [2,15,16,19]. In addition, insufficient awareness of cognitive late effects among patients and their caregivers may further reduce participation, engagement, and long-term adherence to intervention strategies [20,21]. Therefore, there is an urgent need to develop scientifically sound and effective approaches for the management of cognitive symptoms among children and adolescents with cancer [17,22].

Digital health refers to the use of digital technologies to support health care delivery, health management, and rehabilitation, including eHealth, mobile health, and other technology-enabled interventions [23]. Given its accessibility, flexibility, and potential for remote delivery, digital health offers a promising approach to the management of cognitive late effects among children with cancer by facilitating cognitive training, neurocognitive monitoring, education, and supportive care [24-26]. Accordingly, digital health interventions have been increasingly explored to support cognitive management in this population [27]. However, previous studies and reviews have primarily focused on the effectiveness and feasibility of specific digital health modalities, such as computerized cognitive training, for improving cognitive outcomes among children with cancer [27,28]. Consequently, the broader landscape of digital health interventions for cognitive management remains insufficiently characterized, particularly with respect to intervention modalities, delivery approaches, and core functional components. A broader mapping of the existing evidence is therefore needed to better understand how digital health interventions have been applied for cognitive management among children with cancer.

Therefore, this scoping review aimed to map and synthesize the existing evidence on digital health interventions for the management of cognitive late effects among children with cancer, to characterize intervention modalities, delivery approaches, and functional components, and to identify research gaps and priorities for future development. The findings may provide evidence-based insights for optimizing digital health interventions and supporting their clinical translation.

## Methods

### Protocol and Registration

The protocol for this study was registered on the Open Science Framework [29], and the literature search was conducted on August 31, 2026. This scoping review was conducted in accordance with the 5-stage methodological framework proposed by Arksey and O’Malley [30], with the aim of systematically mapping the research landscape of digital health interventions for cognitive management among children with cancer. We specifically included studies that used digital health interventions for cognitive management among children with cancer and examined intervention characteristics, including delivery modalities, functional components, implementation features, and reported outcomes. The reporting of this review was guided by the PRISMA-ScR (Preferred Reporting Items for Systematic Reviews and Meta-Analyses Extension for Scoping Reviews; Checklist 1) to ensure methodological rigor and transparency [31].

### Eligibility Criteria

We used the population-concept-context (PCC) framework instead of the population intervention comparison outcome framework because our research question does not aim to assess a specific outcome or comparison intervention. For this purpose, the PCC framework is recommended by the *JBI Manual for Evidence Synthesis* [32]. In this review, the population comprised children with cancer, and the concept was the use of digital health interventions for cognitive management. Digital health refers to the use of digital technologies to support health care delivery, disease management, health promotion, and health-related outcomes, including mobile health, web-based platforms, computer-assisted programs, and other digital devices. The context was pediatric oncology care and was not operationalized as a separate search category because it was inherently encompassed by the population with childhood cancer.

Included studies were required to fulfill the following criteria.

1. Population: Studies involving individuals diagnosed with cancer before 18 years of age were eligible, without restrictions on sex.
2. Concept: Studies were included if they evaluated digital health approaches for cognitive management in children with pediatric cancer. In this review, digital health-based cognitive management was defined as interventions delivered through mobile devices or related platforms aimed at improving cognitive function or related clinical outcomes. Eligible interventions included those assessing outcomes such as cognitive effectiveness, acceptability, usability, feasibility, cost, or adherence.
3. Context: Studies conducted in any health care or community setting were eligible, and no restrictions were applied regarding the intervention setting.

Eligible study designs were required to include quantitative research. Studies were excluded if they were published study protocols, reviews, editorials, conference proceedings, books, or non-English publications. Studies in which mobile technology was used solely for outcome data collection without providing tailored feedback or counseling based on the collected data were excluded. For interventions involving digital components accessible through apps as well as computers or other devices, studies were included only when participants’ use of digital devices was explicitly reported; otherwise, they were excluded. Studies without accessible full texts were also excluded.

### Information Sources

A systematic search was conducted across multiple electronic databases, including PubMed, Embase (Ovid), CINAHL, PsycINFO, Web of Science, and Cochrane Library, to identify studies published between January 2010 and August 2026. The reference lists of all eligible studies were examined to identify any potentially relevant studies.

### Search

We developed and refined the search strategy iteratively, adapting it as necessary for different databases (see Multimedia Appendix 1 for the full strategy). The PCC framework and corresponding search components used in this study are presented in Table 1.

**Table 1.** Search strategy.

Component number	PCC^a^ framework	Search string
1	P: population	(child OR adolescent* OR teen* OR youth OR minors OR pediatric* OR pediatric OR young OR teenager* OR juvenile* OR “young people” OR “young person*") (neoplasms OR cancer* OR tumor* OR tumor* OR neoplas* OR malignan* OR oncology OR leukemia* OR leukemia* OR lymphoma* OR sarcoma* OR carcinoma* OR neuroblastoma* OR astrocytoma* OR glioma* OR medulloblastoma)
2	C: concept	(telemedicine OR mobile apps OR internet OR mHealth OR eHealth OR “digital health” OR telehealth OR web-based OR online OR internet-based OR “digital intervention” OR computerized OR computer-based OR home-based OR “remote intervention” OR “remote delivery” OR “remote monitoring” OR smartphone* OR (mobile AND app) OR (mobile AND app*)) (Cognition OR “Cognitive Dysfunction” OR “Cognitive Training” OR Memory OR Attention OR “Executive Function” OR cognit* OR neurocognit* OR neuropsycholog* OR “cognitive function” OR “cognitive impairment” OR “cognitive disorder” OR “cognitive training” OR “cognitive rehabilitation” OR “cognitive remediation” OR “brain training” OR memory OR “working memory” OR attention OR attentional OR “executive function” OR “cognitive control” OR “processing speed” OR “psychomotor speed”)
3	C: context	—^b^

a PCC: population-concept-context.

b Not available.

### Selection of Sources of Evidence

Study selection was independently conducted by 2 reviewers (TL and MT), who screened titles, abstracts, and full texts according to the predefined eligibility criteria. Disagreements were resolved through discussion, and a third reviewer (LM) was consulted when consensus could not be reached.

### Data Charting and Synthesis of Results

Data were extracted and charted using Microsoft Excel. Extracted data included publication year, study design, participant characteristics, cancer types, digital health modalities, intervention characteristics, and reported outcomes. Data related to intervention functions, cognitive management strategies, and implementation features were also extracted.

Data synthesis was conducted through an iterative process of summarization, coding, and comparison. Descriptive analysis was used to summarize the characteristics of included studies. A thematic content analysis approach was applied to synthesize the functional components and key characteristics of digital health cognitive management interventions [33]. Following established principles of qualitative content analysis [34], extracted data related to intervention functions, cognitive management strategies, and implementation features were systematically coded and categorized. Similar codes were grouped into broader categories, and key themes were generated through iterative comparison across studies.

### Critical Appraisal of Individual Sources of Evidence

The methodological quality of the included studies was assessed using the 2018 version of the Mixed Methods Appraisal Tool by the same 2 reviewers (TL and MT). Any disagreements during the appraisal process were resolved through discussion until consensus was achieved.

## Results

### Basic Characteristics of the Included Studies

A total of 5371 records were identified through database searches. After removing duplicates and other ineligible sources, 2495 titles and abstracts were screened for eligibility, and 279 full-text articles were subsequently assessed. Ultimately, 19 publications [24,35-52] representing 14 independent study samples were included in this review (Figure 1). No additional eligible studies were identified through backward citation searching. No new studies were identified from the updated search.

**Figure 1.**
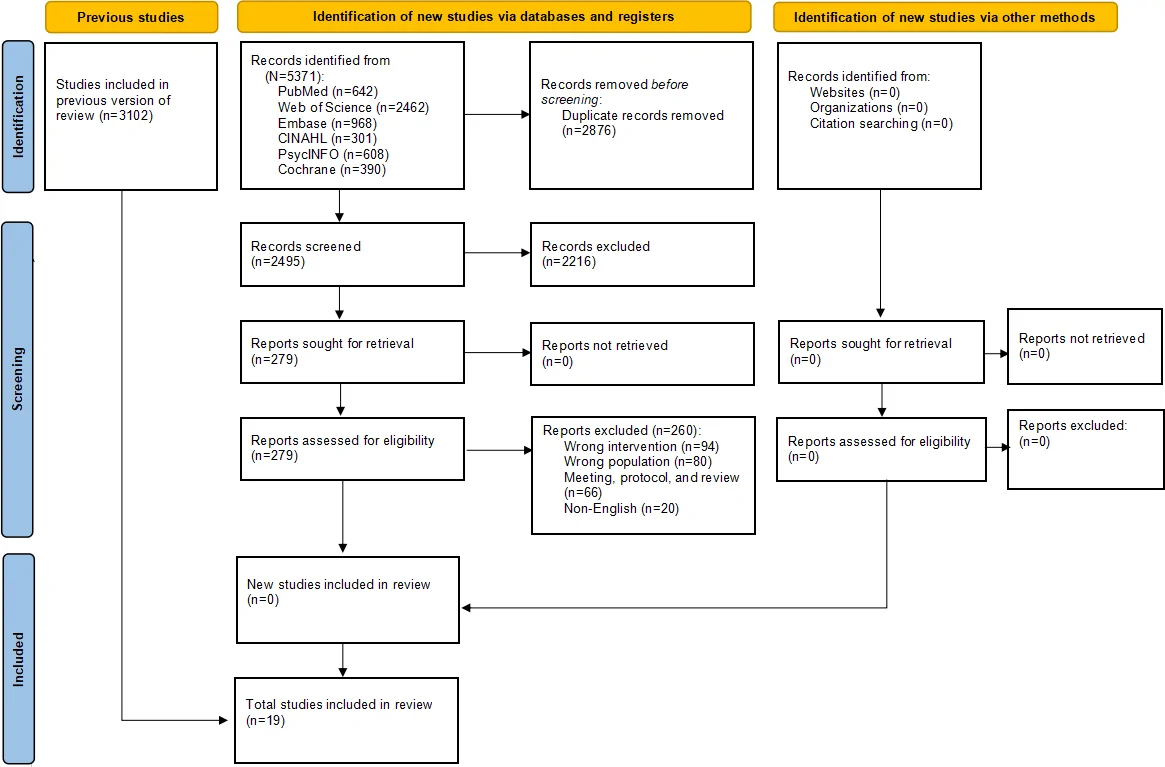
PRISMA (Preferred Reporting Items for Systematic Reviews and Meta-Analyses) study screening flowchart.

Several included publications represented multiple reports from the same underlying studies. Specifically, 5 publications [24,35-38] originated from the same randomized controlled trial (RCT) involving 68 survivors of childhood cancer but reported different aspects of the intervention, including efficacy, feasibility, coaching conditions, transfer effects, and caregiver perspectives. Additionally, 2 publications [39,40] were derived from the same Nintendo Wii intervention study involving 13 participants but focused on different outcomes. These publications were retained as separate reports because they addressed distinct research questions; however, they were considered as a single independent sample when summarizing study characteristics and calculating the overall sample size. The sample size reported by Scott et al [38] was not counted separately because this study focused on caregiver perspectives rather than providing an independent patient sample. After accounting for overlapping samples, the 19 publications represented 14 independent study samples involving 566 children with cancer.

Among the 19 included publications, RCTs were the predominant study design (n=14), including one randomized crossover controlled trial. Two studies used quasi-experimental designs, while one study adopted a quantitative descriptive design, one used a pilot before-and-after design, and one applied a mixed methods design.

From a temporal perspective, the included studies were primarily published between 2010 and 2022 (Figure 2). In terms of geographical distribution, the included studies were predominantly conducted in North America and Europe. The majority originated from the United States (13/19) [24, 35-38, 42-44, 46-49, 52], followed by Sweden (2/19) [39, 40], with one study each from Canada [45], the Netherlands [50], Switzerland [41], and Estonia [51]. Overall, the evidence base is concentrated in North America and Europe, with limited representation from other world regions.

**Figure 2.**
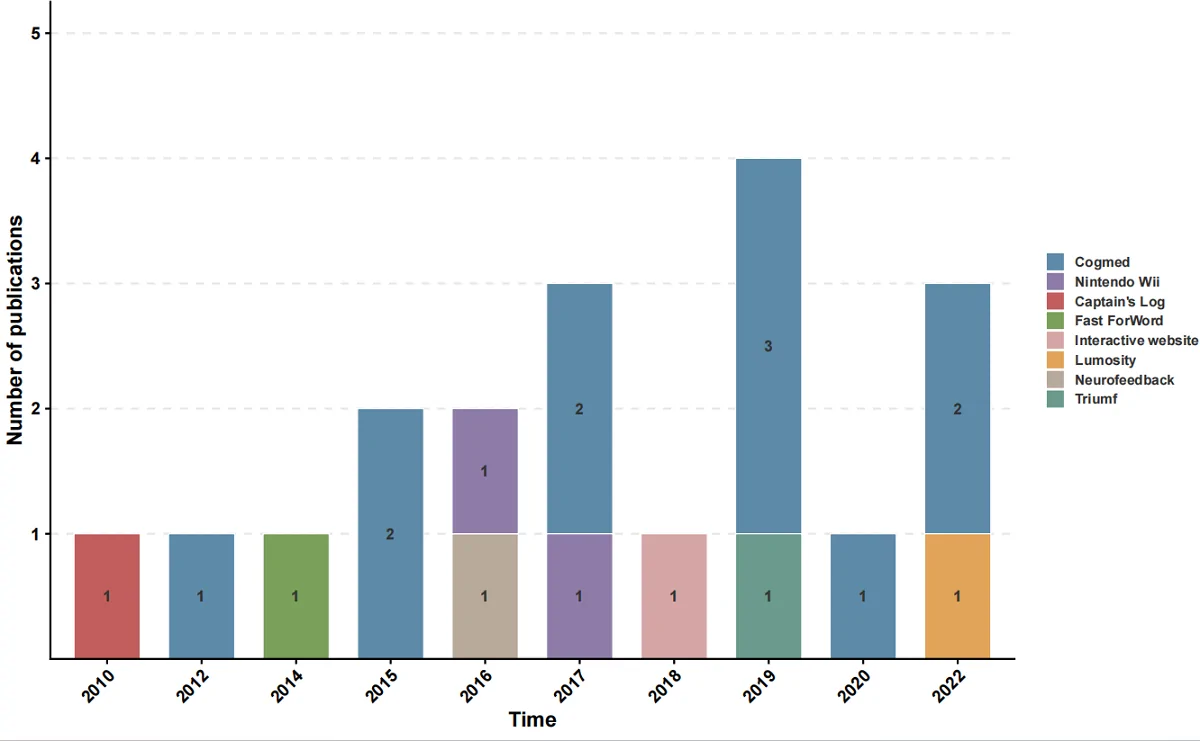
Temporal distribution of the included studies by publication year and digital health intervention type [24, 35–52].

In terms of delivery modes, digital health interventions were predominantly implemented through computer-based cognitive training programs, with other approaches including internet-based physical activity, mobile apps, and electroencephalography (EEG)-based neurofeedback. Among these, computerized cognitive training systems (eg, Cogmed, provided by Pearson) represented the most commonly used intervention, accounting for a substantial proportion of the included studies (11/19) [24,35-38,41-46]. These interventions were typically delivered via computers or tablets, often combined with remote guidance or home-based training. In addition to Cogmed, other software-based interventions were also identified, including programs such as Fast ForWord (Scientific Learning Corporation) [47], Captain’s Log (BrainTrain, Inc) [48], and Lumosity (Lumos Labs, Inc) [49], which similarly rely on computers or mobile devices to deliver cognitive training content. Moreover, one study [50] used neurofeedback interventions, which use computer interfaces to provide real-time feedback on brain activity to facilitate cognitive regulation.

A subset of studies has begun to explore game-based and gamified intervention approaches, such as Nintendo Wii/Wii-Fit exergaming [39,40] and newly developed digital health gaming applications (eg, Triumf) [51]. These approaches integrate physical activity or entertainment elements into the intervention to enhance engagement and adherence among children. Additionally, one study [52] used web-based platforms or interactive websites to deliver interventions, thereby expanding the application scenarios of digital health.

Regarding implementation-related outcomes, most studies focused on cognitive-related outcomes, including working memory, attention, executive function, processing speed, and intelligence. Some studies additionally examined academic achievement [36,42,45,47] and serum brain-derived neurotrophic factor (BDNF) levels [49]. In addition, most studies evaluated the feasibility of the interventions [35,42-49,51], acceptability [35,36,42,44,48,51], and adherence [35,36,43,45], while only a limited number of studies assessed adverse events as an outcome measure [43,46]. Economic outcomes were not reported in the included studies.

The results of the quality assessment showed that most studies were of good quality. Specific details of the quality assessment can be found in Multimedia Appendix 2. Table 2 provides an overview of the key characteristics of the included studies.

**Table 2.** Summary of basic characteristics of the included studies.

Author and year	Country	Aim of the study	Study design	Cancer type	Treatment modality	Total sample (group-specific sample sizes)	Age range (y)	Nature of digital health	Outcomes measured	Results (summary of findings)
Benzing et al [41], 2020	Switzerland	To compare the effects of digital health interventions on cognitive function.	RCT^a^	Leukemia and lymphomas (n=38); CNS^b^ tumors and neuroblastomas (n=16); other cancer diagnoses (n=15)	Chemotherapy + radiotherapy + surgery	Total: 69 (Cogmed group: n=23; exergaming group: n=22; control group: n=24)	7‐16	Cogmed	Executive function with the Children’s Working Memory Test Package and BRIEF-P^c^; language working memory, planning ability, and language memory with KABC-II^d^; attention and processing speed with WISC-IV^e^	Compared with the control group, visual working memory improved in the participants in the Cogmed group (*P*=.01).
Carlson-Green et al [42], 2017	America	To determine the feasibility and effectiveness of digital health cognitive interventions.	A pilot study: a before and after study	Brain tumor (N=20)	—^f^	Total: 20	8-18	Cogmed	Working memory with Automated Working Memory Assessment; academic achievement with WJ-III^g^; executive function with BRIEF-P; satisfaction	Improved working memory and mathematics performance at posttraining and follow-up; improved parent-reported executive function; extended training showed no additional working memory benefits.
Conklin et al [24], 2015	America	To examine the short-term efficacy of digital cognitive interventions and the neural correlates associated with cognitive changes.	RCT	ALL^h^ (n=47), brain tumor (n=21)	Chemotherapy + radiotherapy + surgery	Total: 68 (experimental group: n=34; control group: n=34)	8‐16	Cogmed	Working memory with WISC-IV; continuous attention with Conners’ Continuous Performance Test II; academic achievement with WJ-III; attention with Conners-3^i^; executive function with BRIEF-P	Improved working memory, executive function, attention, processing speed, and memory compared with controls; reduced activation in the left prefrontal cortex and bilateral medial frontal cortex after training.
Cox et al [35], 2015	America	To assess the feasibility and acceptability of remote computerized interventions.	RCT	ALL (n=47), brain tumor (n=21)	Chemotherapy + radiotherapy	Total: 68 (experimental group: n=34; control group: n=34)	8‐16	Cogmed	Intelligence test with the Wechsler Abbreviated Scale of Intelligence, computer literacy, satisfaction, and compliance	A total of 30 (88%) participants completed the intervention. Caregivers reported that they usually had the time to complete the training (63%), considered the training beneficial (70%), and would recommend this intervention to others (93%).
Fournier-Goodnight et al [36], 2019	America	To examine adherence, satisfaction, and efficacy of Cogmed across different coaching conditions.	RCT	ALL (n=47), brain tumor (n=21)	Chemotherapy + radiotherapy + surgery	Total: 68 (experimental group: n=34; control group: n=34)	8‐16	Cogmed	Working memory with WISC-IV; processing speed and attention with Conners’ Continuous Performance Test II and Conners-3; academic achievement with WJ-III	Cogmed was perceived as practical and feasible; equivalence was supported in attention, working memory, processing speed, and academic fluency outcomes, with maintained coach adherence.
Hardy et al [43], 2012	America	To examine the feasibility and preliminary efficacy of home-based digital health programs.	RCT	ALL (n=11), medulloblastoma/PNET^j^ (n=4), ependymoma (n=3), other cancer diagnoses (n=2)	Chemotherapy + radiotherapy + surgery	Total: 20 (experimental group: n=13; control group: n=7)	8‐16	Cogmed	Working memory with WRAML2^k^; attention with Conners-3; adverse events with the side effect scoring scale SERS^l^; feasibility	High adherence (85%) with no adverse events; improved visual working memory and parent-rated learning problems compared with active controls, but no significant difference in verbal working memory.
Hocking et al [44], 2019	America	To evaluate the feasibility and acceptability of digital health interventions.	RCT	Brain tumor (N=27)	Chemotherapy + radiotherapy + surgery	Total: 27 (experimental group: n=14; control group: n=13)	7‐16	Cogmed	Working memory; processing speed with WISC-IV; executive function, attention using CBCL^m^; feasibility, acceptability	A total of 14 out of 27 participants completed at least 20 training sessions; completers showed better baseline auditory attention, while parents reported satisfaction, frustration, and completion barriers.
Mendoza et al [37], 2019	America	To investigate whether cognitive improvements following digital health interventions generalize to social functioning outcomes	RCT	ALL (n=47), brain tumor (n=21)	Chemotherapy + radiotherapy	Total: 68 (experimental group: n=34; control group: n=34)	8‐16	Cogmed	Attention with Conners-3; working memory with WISC-IV	Greater improvements in attention and working memory compared with controls; computerized cognitive training effects did not generalize to social functioning.
Peterson et al [45], 2022	Canada	To evaluate the feasibility of digital health cognitive interventions compared with standard mathematics interventions	RCT	Brain tumor (N=28)	Radiotherapy	Total: 28 (Cogmed group: n=10; JumpMath group: n=9; control group: n=9)	7‐17	Cogmed	Mathematical calculation and reasoning with math calculation and math fluency subtests; working memory with WISC-IV, adherence, and feasibility.	High parent satisfaction (94%) and perceived feasibility; improved auditory working memory compared with controls, with additional gains in visual working memory.
Siciliano et al [46], 2022	America	To evaluate the feasibility and preliminary effects of digital cognitive interventions	RCT	Brain tumor (N=41)	Chemotherapy + radiotherapy + surgery	Total: 41 (experimental group: n=20; control group: n=21	8‐16	Cogmed	Working memory with WRAML2; attention with Conners-3; adverse events with SERS; feasibility	Participants completed half of the sessions; Working Memory Index and Neurocognitive Test Battery scores improved immediately post intervention but were not maintained at follow-up, with preliminary improvements in parent-reported executive function.
Scott et al [38], 2017	America	To investigate caregiver responses following digital health interventions	Quantitative study	ALL (n=25), brain tumor (n=18)	—	A total of 43 children and their caregivers (from 68 survivors of childhood cancer)	8‐16	Cogmed	Clarity of the intervention and understanding of the content	Caregivers reported high perceived importance, usefulness, clarity, and relevance of results; most were satisfied (84%), with some concerns about long-term impacts (25%). Preferred result delivery methods were mailed letters and emails.
Hardy et al [48], 2010	America	To evaluate the feasibility of digital health programs	Quasi-experimental	ALL (n=3), medulloblastoma/primitive neuroectodermal tumor (n=4), ependymoma (n=2)	Chemotherapy + radiotherapy + surgery	Total: 18 (experimental group: n=9; control group: n=9)	10‐17	Captain’s Log	Working memory with WISC-IV; attention with Conners-3; feasibility, acceptability	Improved working memory and reduced parent-reported attention problems after intervention.
Gooch et al [49], 2022	America	To investigate the feasibility of digital health-based cognitive rehabilitation interventions	Quasi-experimental	Stepsarcoma (n=3), mixed germ cell tumor (n=3), ALL (n=3), others (n=4)	—	Total: 14 (experimental group: n=7; control group: n=7)	12‐25	Lumosity	Neurocognitive test battery (CogState Ltd) and serum BDNF^n^ levels; feasibility	High feasibility with 71% intervention adherence; the nonintervention group showed worsening cognitive impairment in processing speed, visual attention, attention/working memory, and executive function, with no significant changes in serum BDNF levels.
De Ruiter et al [50], 2016	Netherlands	To examine the effects of computerized neurofeedback training on cognitive function	RCT	Brain tumor (N=80)	Chemotherapy + radiotherapy + surgery	Total: 80 (experimental group: n=40; control group: n=40)	8‐18	Neurofeedback	Attention with the Attention Network Test; executive functioning with WISC-IV and BRIEF-P	Similar improvements were observed in both groups; neurofeedback showed no specific neurocognitive treatment effect in survivors of pediatric brain tumors.
Sabel et al [39], 2017	Sweden	To evaluate the effects of app-based physical activity interventions	RCT	Brain tumor (N=13)	Chemotherapy + radiotherapy + surgery	Total: 13 (experimental group: n=7; control group: n=6)	7‐17	Nintendo Wii	Cognitive function with map tasks and Rey normative tests; executive function with the Assessment of Motor and Process Skills	All participants completed the program; active video gaming significantly improved assessment of motor and process skills compared with baseline.
Sabel et al [40], 2016	Sweden	To investigate whether internet-based serious games can improve physical and cognitive functioning	A randomized crossover controlled trial	Brain tumor (N=13)	Chemotherapy + radiotherapy + surgery	Total: 13	7‐17	Nintendo Wii	Body coordination with BOT-2^o^	Active video gaming significantly improved body coordination and bilateral coordination, while no significant changes were observed in other BOT-2 domains.
Howell et al [52], 2018	America	To evaluate the effectiveness of web-based physical activity interventions	RCT	ALL (n=18), acute myeloid leukemia (n=1), CNS tumors (n=17), others (n=42)	—	Total: 78 (experimental group: n=53; control group: n=25)	11‐15	Interactive website	Cognitive flexibility with the Delis-Kaplan Executive Function System	The intervention group showed improvements in physical fitness, neurocognitive function, and HRQOL^p^ compared with controls.
Tark et al [51], 2019	Estonia	To assess the usability, acceptability, and feasibility of app-based interventions	Mixed methods	CNS tumor (n=1), Leukemia (n=6), other (n=2)	—	Total: 9	7‐14	Triumf (A newly developed mobile health game)	Content of app feasibility and acceptability	High usability and acceptability were reported; 67% of participants continued using the game after the study. Psychological support and educational modules were frequently used. The intervention reduced overall health problems (*P*=.003) and showed trends toward improved depression and anxiety symptoms.
Palmer et al [47], 2014	America	To investigate the feasibility of digital health-based cognitive management interventions	RCT	Brain tumor	—	Total: 81 (experimental group: n=43; control group: n=38)	4‐21	Fast ForWord	Reading ability with WJ-III	A total of 39.5% of participants completed 30 training sessions; females showed longer training durations than males. No significant differences in reading outcomes were found between randomized groups.

a RCT: randomized controlled trial.

b CNS: central nervous system.

c BRIEF-P: Behavior Rating Inventory of Executive Function–Preschool Version.

d KABC-II: Kaufman Assessment Battery for Children–Second Edition.

e WISC-IV: Wechsler Intelligence Scale for Children–Fourth Edition.

f Not available.

g WJ-Ⅲ: Woodcock-Johnson Tests of Achievement–Third Edition.

h ALL: acute lymphoblastic leukemia.

i Conners-3: Conners Third Edition.

j PNET: primitive neuroectodermal tumor.

k WRAML2: Wide Range Assessment of Memory and Learning–Second Edition.

l SERS: Side Effect Rating Scale.

m CBCL: Child Behavior Checklist.

n BDNF: brain-derived neurotrophic factor.

o BOT-2: Bruininks-Oseretsky Test of Motor Proficiency, Second Edition.

p HRQOL: health-related quality of life.

### Digital Health Intervention Program for Cognitive Management

Most included studies (14/19) used computer-based digital health interventions for cognitive management among children with cancer, with Cogmed being the most frequently used platform (11/19). Other delivery modalities included internet-based physical activity interventions, mobile app-based interventions, and EEG-based neurofeedback training. Regarding target cognitive domains, existing interventions predominantly focused on specific cognitive functions, with working memory and attention being the most frequently targeted domains. All digital health intervention models included cognitive training components aimed at improving cognitive function, whereas only a limited number of studies integrated additional supportive functions, such as coaching support, activity monitoring, educational materials, and screening functions. The details can be found in Table 3. Figure 3 presents the evidence gap map of cognitive management across different digital health intervention modalities among children with cancer.

**Table 3.** Content of digital health intervention measures.

Classification of digital health	Delivery modality	Number of included studies (n)	Target cognitive domain	Function and intervention contents
Cogmed	Computer-based cognitive training	11	Working memory	Coach-supported and adaptive difficulty working memory training
Captain’s Log	Computer-based cognitive training	1	Attention and basic problem-solving/reasoning skills	Cognitive training and telephone communication
Lumosity	Computer-based cognitive training	1	Memory and attention	Cognitive training
Fast ForWord	Computer-based reading training	1	Reading decoding ability	Cognitive training
Nintendo Wii	Internet-based physical activity	2	Executive function and attention	Activity monitoring, coaching support, and active video gaming to promote cognitive and motor function
Interactive website	Internet-based physical activity	1	Intelligence and cognitive flexibility	Activity monitoring, online cognitively engaging physical training, and educational materials
Triumf	Mobile app	1	Attention	Gamified cognitive training, supportive functions, educational functions, and screening functions
Neurofeedback	EEG^a^-based neurofeedback training	1	Cognitive function	Enhancement of cognitive function through EEG-based self-regulation of brain activity

a EEG: electroencephalography.

**Figure 3.**
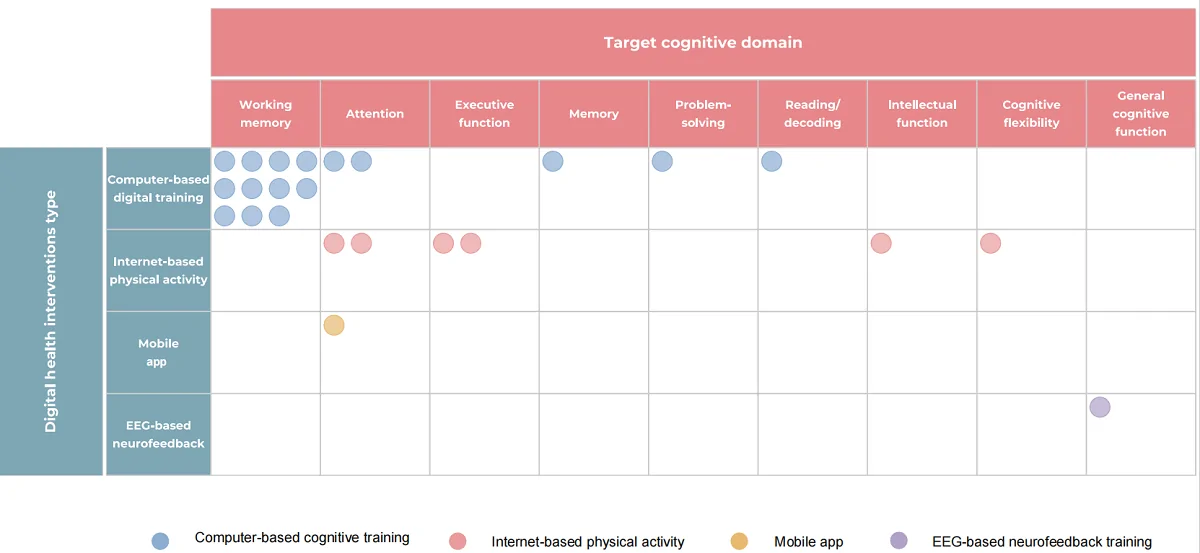
Gap map of cognitive management across different digital health intervention modalities among children with cancer. EEG: electroencephalography.

## Discussion

### Principal Finding

This review synthesized evidence from 19 publications representing 14 independent study samples published between 2011 and 2022 and characterized the intervention modalities, functional components, implementation features, and research gaps of digital health interventions for cognitive management in populations with pediatric cancer [24,34-52]. Our findings highlight substantial heterogeneity in intervention design, participant characteristics, and outcome measures across studies examining digital health interventions for cognitive management among children with cancer. The findings indicate that most of the included studies (11/19) reported implementation-related outcomes [35,36,42-49,51], such as feasibility, acceptability, and adherence, providing preliminary evidence regarding the implementation potential of digital health interventions for cognitive management among children with cancer. However, the evidence base remains limited and requires further development.

### Current Landscape of Digital Health Interventions for Cognitive Management

In recent years, digital health technologies have rapidly advanced in the field of cognitive management, particularly among populations such as older adults with mild cognitive impairment, where diverse application models have been developed. Previous studies have demonstrated that digital health interventions have expanded beyond traditional computerized cognitive training to encompass a variety of approaches, including computerized cognitive training, physical activity-based interventions, serious games, and remote monitoring technologies, reflecting increasing diversity in intervention design [53,54]. In contrast, the application of digital health interventions for cognitive management among children with cancer remains at an early stage of development. The findings of this review indicate that digital health interventions in this population remain relatively limited, with most studies focusing on computer-based cognitive training and remotely delivered intervention models. Among these, programs for adaptive working memory training, such as Cogmed, were the most frequently adopted intervention approaches. This finding is consistent with previous literature, indicating that digital cognitive interventions for children with cancer have predominantly focused on single-domain computerized or application-based cognitive training approaches [28,43]. Furthermore, evidence from both the included studies and previous research suggests that outcome assessments in digital health-based cognitive interventions remain relatively narrow [28,46], primarily focusing on changes in specific cognitive domains, particularly working memory. The observed intervention benefits have mainly been limited to near-transfer effects, referring to improvements in trained tasks or closely related cognitive abilities, whereas evidence regarding far-transfer effects, such as improvements in academic performance and daily functioning, remains inconsistent [55,56]. Several factors may contribute to these limitations. First, cognitive impairment in children with cancer is multidimensional, involving multiple cognitive domains, including executive function, memory, attention, and processing speed. Therefore, interventions targeting a single cognitive domain may improve specific cognitive skills but may be insufficient to address broader cognitive and functional difficulties [17,57]. Second, children with cancer represent a heterogeneous population, with variations in developmental stage, cognitive abilities, cancer type, and treatment history. Such heterogeneity may contribute to differences in cognitive impairment patterns and intervention responses, thereby increasing the complexity of evaluating intervention effects [15]. In addition, limited far-transfer effects may be related to the mismatch between current digital health intervention targets and the broader functional needs of children with cancer. Most existing interventions primarily focus on improving specific cognitive abilities, such as working memory; however, whether cognitive improvements achieved through training can translate into benefits in broader functional domains, including academic performance, daily functioning, and social adaptation, remains influenced by multiple factors [17,58].

This review further explored the functional characteristics of digital health interventions for cognitive management among children with cancer. The findings indicated that existing interventions remain primarily focused on cognitive training, whereas the integration of additional functions, including educational support, behavioral management, psychosocial support, and family engagement, remains limited. In contrast, digital health applications in chronic disease management and mental health fields have increasingly adopted multifunctional intervention models by integrating components such as health education, symptom monitoring, behavioral interventions, emotional support, and family involvement to establish more comprehensive support systems [59,60]. Such integrated designs have demonstrated potential for enhancing user engagement and promoting long-term adherence [61,62]. The limited functional integration observed in pediatric cancer cognitive management may reflect the early developmental stage of this field. Most existing interventions have been adapted from established cognitive training platforms, and their functional scope therefore remains largely restricted to cognitive improvement. However, children with cancer often have broader supportive needs related to disease knowledge, emotional adjustment, social adaptation, and self-management [63,64]. Therefore, relying solely on isolated cognitive training may not adequately address their comprehensive needs [65,66].

Future digital health interventions for cognitive management among children with cancer should move beyond single-domain cognitive training toward integrated, multidimensional intervention strategies. From a functional design perspective, future systems should incorporate multiple cognitive domains, including attention, working memory, executive function, and processing speed, while integrating additional components such as health education, behavioral support, psychosocial interventions, and symptom management to address the multidimensional needs associated with cancer-related cognitive impairment and disease management [65,66]. Considering variations in children’s age, cognitive development, and disease status, modular and adaptive designs may be adopted to dynamically tailor intervention content and difficulty according to individual characteristics [15]. For younger children, greater emphasis may be placed on gamified designs and caregiver-assisted functions, whereas older children and adolescents may benefit from enhanced disease education, self-management support, and psychosocial functions. In addition, greater involvement of caregivers and health care professionals through training assistance, symptom feedback, professional guidance, and remote monitoring may improve intervention acceptability and long-term adherence [65,67]. Furthermore, digital health interventions should be integrated into pediatric oncology care pathways, allowing initiation during hospitalization and continued implementation in home settings. Future studies should use large-scale, multicenter RCTs with age-stratified designs and long-term follow-up to further evaluate the effects of multidomain digital health interventions on core cognitive outcomes as well as far-transfer outcomes, including academic performance, daily functioning, and quality of life, thereby facilitating clinical translation [65,68].

### Evidence Gaps and Future Research Priorities

Among the included studies, only a few reported adverse events as safety outcomes [43,46], suggesting that safety monitoring of digital health interventions for cognitive management among children with cancer remains insufficient. Therefore, future research should incorporate safety assessment as a core outcome measure and establish standardized reporting guidelines for adverse events to improve the quality of evidence and support clinical translation. Beyond safety considerations, broader implementation-related evidence, including economic feasibility, is also essential for translating digital health interventions into routine clinical practice [68]. However, no studies included in this review evaluated the economic aspects of digital health-based cognitive management among children with cancer. Future research should incorporate health economic evaluations to assess the feasibility, sustainability, and scalability of these interventions, thereby informing policy development and clinical decision-making. Finally, most of the included studies were RCTs focusing primarily on intervention efficacy, whereas qualitative studies and process evaluations were relatively limited. This restricts a deeper understanding of adherence, user experience, and implementation barriers. Previous research has also suggested that the long-term effects of cognitive training may be influenced by adherence and contextual factors. Therefore, in addition to conducting high-quality RCTs, future studies should incorporate qualitative or mixed methods designs and strengthen evaluations of acceptability, feasibility, and implementation processes to optimize intervention design and facilitate clinical translation.

### Strengths and Limitations

This study has several strengths. First, it specifically focused on the population with pediatric cancer and synthesized evidence from diverse study designs, providing a comprehensive overview of digital health interventions for cognitive management in children with cancer. The findings characterize intervention modalities, delivery approaches, and functional components of existing digital health interventions and provide valuable insights for future intervention development. This review also highlights current evidence gaps and emerging research priorities, including the need for optimized functional module design, improved user experience, and integration of clinically relevant outcomes and assessment tools. These findings may support researchers and clinicians in advancing the development and implementation of digital health interventions in pediatric oncology.

There are also some limitations. First, the broad scope and complexity of the search strategy may have led to the omission of relevant studies, despite adherence to the PRISMA-ScR guidelines to enhance rigor and transparency. Second, the heterogeneity of cognitive outcome measures, including the combination of subjective and objective assessments, limited data synthesis and reduced comparability across studies. Third, the heterogeneity in participant age ranges across the included studies is another limitation. Some studies included participants older than 18 years, while age-disaggregated data were not consistently available. Therefore, findings from mixed-age samples should be interpreted cautiously when applied specifically to children and adolescents. Fourth, only English-language publications were included, and most studies were conducted in North America and Europe, which may limit the generalizability of the findings to other regions. Therefore, future research should expand search strategies and database coverage, include studies published in other languages, and incorporate more diverse populations across different continents to improve the comprehensiveness and global applicability of the evidence base.

### Conclusion

This scoping review synthesized the existing evidence on digital health interventions for cognitive management among children with cancer. The findings indicate that this field remains in an early stage of development, with current interventions primarily focusing on cognitive training delivered through computer-based programs and other digital platforms. Although these interventions show potential for supporting cognitive function, most approaches remain centered on single-domain training, with limited integration of supportive components and relatively narrow outcome assessments. Future research should prioritize the development of multidimensional, personalized, and integrated digital health interventions that incorporate cognitive training, supportive functions, and clinically relevant outcomes. High-quality multicenter RCTs, together with safety monitoring, health economic evaluations, and qualitative or mixed-methods studies, are needed to strengthen the evidence base and facilitate clinical implementation.

## Supplementary material

10.2196/104881 Multimedia Appendix 1 Pubmed search strategy.

10.2196/104881 Multimedia Appendix 2 Critical appraisal of the selected studies using the Mixed Methods Appraisal Tool.

10.2196/104881 Checklist 1 PRISMA checklist.

## Abbreviations

BDNF: brain-derived neurotrophic factor
PCC: population-concept-context
PRISMA-SrC: Preferred Reporting Items for Systematic Reviews and Meta-Analyses Extension for Scoping Reviews
RCT: randomized controlled trial
