# Supplementary material for: Application of Digital Health Interventions for Cognitive Management in Children With Cancer: Scoping Review
Source: J Med Internet Res. 2026 Nov 5;28:e104881. doi: 10.2196/104881 (PMC13739209; doi:10.2196/104881)
Supplement: Supplementary file 2 [file jmir-v28-e104881-s001.docx]

# PubMed search strategy

| **PubMed** | | |
| --- | --- | --- |
| #5 | #1 AND #2 AND #3 AND #4 Filters: **Publication date from 2010/01/01 to 2026/08/31; Humans** | 642 |
| #4 | Search (((((("Telemedicine"[MeSH Terms]) OR ("Mobile Applications"[MeSH Terms])) OR ("Internet"[MeSH Terms])) OR (mhealth[Title/Abstract] OR ehealth[Title/Abstract] OR "digital health"[Title/Abstract] OR telemedicine[Title/Abstract] OR telehealth[Title/Abstract] OR “web-based”[Title/Abstract] OR online[Title/Abstract] OR “internet-based”[Title/Abstract] OR "digital intervention"[Title/Abstract] OR computerized[Title/Abstract] OR computer-based[Title/Abstract] OR home-based[Title/Abstract] OR "remote intervention"[Title/Abstract]OR "remote delivery"[Title/Abstract] OR "remote monitoring"[Title/Abstract] OR smartphone*[Title/Abstract])) OR (mobile[Title/Abstract] AND app[Title/Abstract])) OR (mobile[Title/Abstract] AND application*[Title/Abstract])) Sort by: Author | 735,580 |
| #3 | Search (((("Child"[MeSH Terms]) OR ("Adolescent"[MeSH Terms])) OR ("Minors"[MeSH Terms])) OR (Child*[Title/Abstract] OR Adolescent*[Title/Abstract] OR Teen*[Title/Abstract] OR Youth[Title/Abstract] OR Minors[Title/Abstract] OR Pediatric*[Title/Abstract] OR Paediatric[Title/Abstract] OR Young[Title/Abstract] OR Teenager*[Title/Abstract] OR Juvenile*[Title/Abstract] OR "Young People"[Title/Abstract] OR "Young Person*"[Title/Abstract])) Sort by: Author | 4,943,075 |
| #2 | Search (("Neoplasms"[MeSH Terms]) OR (Cancer*[Title/Abstract] OR Tumor*[Title/Abstract] OR Tumour*[Title/Abstract] OR Neoplas*[Title/Abstract] OR Malignan*[Title/Abstract] OR Leukemia*[Title/Abstract] OR Leukaemia*[Title/Abstract] OR Lymphoma*[Title/Abstract] OR Sarcoma*[Title/Abstract] OR Carcinoma*[Title/Abstract] OR Neuroblastoma*[Title/Abstract] OR Astrocytoma*[Title/Abstract] OR Glioma*[Title/Abstract] OR Medulloblastoma*[Title/Abstract])) Sort by: Author | 5,997,475 |
| #1 | Search (((((((("Cognition"[MeSH Terms]) OR ("Cognitive Dysfunction"[MeSH Terms])) OR ("Cognitive Training"[MeSH Terms])) OR ("Memory"[MeSH Terms]))  OR ("Attention"[MeSH Terms])) OR ("Executive Function"[MeSH Terms]))  OR (cognit*[Title/Abstract] OR neurocognit*[Title/Abstract] OR neuropsycholog*[Title/Abstract] OR "cognitive function"[Title/Abstract] OR "cognitive impairment"[Title/Abstract] OR "cognitive disorder"[Title/Abstract] OR "cognitive training"[Title/Abstract] OR "cognitive rehabilitation"[Title/Abstract]  OR "cognitive remediation"[Title/Abstract] OR "brain training"[Title/Abstract] OR memory[Title/Abstract] OR "working memory"[Title/Abstract] OR attention[Title/Abstract] OR attentional[Title/Abstract] OR "executive function"[Title/Abstract] OR "cognitive control"[Title/Abstract] OR "processing speed"[Title/Abstract] OR "psychomotor speed"[Title/Abstract]))) Sort by: Author | 1,774,836 |

Date of search: 2026.8.31
